# Nature-Inspired Search Method and Custom Waste Object Detection and Classification Model for Smart Waste Bin

**DOI:** 10.3390/s22166176

**Published:** 2022-08-18

**Authors:** Israel Edem Agbehadji, Abdultaofeek Abayomi, Khac-Hoai Nam Bui, Richard C. Millham, Emmanuel Freeman

**Affiliations:** 1 Honorary Research Associate, Faculty of Accounting and Informatics, Durban University of Technology, P.O. Box 1334, Durban 4000, South Africa; 2 Department of Information and Communication Technology, Mangosuthu University of Technology, P.O. Box 12363, Durban 4026, South Africa; 3 Supercomputing Application Center, Korea Institute of Science and Technology Information (KISTI), Daejeon 34141, Korea; 4 ICT and Society Research Group, Department of Information Technology, Durban University of Technology, P.O. Box 1334, Durban 4000, South Africa; 5 Department of Computer Science, Ghana Communication Technology University, Accra PMB 100, Ghana

**Keywords:** smart bin, Internet of Things (IoT) enabled, object detection and classification, convolutional neural network (CNN), You-Only-Look-Once (YOLO), Kestrel-based search algorithm (KSA)

## Abstract

Waste management is one of the challenges facing countries globally, leading to the need for innovative ways to design and operationalize smart waste bins for effective waste collection and management. The inability of extant waste bins to facilitate sorting of solid waste at the point of collection and the attendant impact on waste management process is the motivation for this study. The South African University of Technology (SAUoT) is used as a case study because solid waste management is an aspect where SAUoT is exerting an impact by leveraging emerging technologies. In this article, a convolutional neural network (CNN) based model called You-Only-Look-Once (YOLO) is employed as the object detection algorithm to facilitate the classification of waste according to various categories at the point of waste collection. Additionally, a nature-inspired search method is used as learning rate for the CNN model. The custom YOLO model was developed for waste object detection, trained with different weights and backbones, namely darknet53.conv.74, darknet19_448.conv.23, Yolov4.conv.137 and Yolov4-tiny.conv.29, respectively, for Yolov3, Yolov3-tiny, Yolov4 and Yolov4-tiny models. Eight (8) classes of waste and a total of 3171 waste images are used. The performance of YOLO models is considered in terms of accuracy of prediction (Average Precision—AP) and speed of prediction measured in milliseconds. A lower loss value out of a percentage shows a higher performance of prediction and a lower value on speed of prediction. The results of the experiment show that Yolov3 has better accuracy of prediction as compared with Yolov3-tiny, Yolov4 and Yolov4-tiny. Although the Yolov3-tiny is quick at predicting waste objects, the accuracy of its prediction is limited. The mean AP (%) for each trained version of YOLO models is Yolov3 (80%), Yolov4-tiny (74%), Yolov3-tiny (57%) and Yolov4 (41%). This result of mAP (%) indicates that the Yolov3 model produces the best performance results (80%). In this regard, it is useful to implement a model that ensures accurate prediction to develop a smart waste bin system at the institution. The experimental results show the combination of KSA learning rate parameter of 0.0007 and Yolov3 is identified as the accurate model for waste object detection and classification. The use of nature-inspired search methods, such as the Kestrel-based Search Algorithm (KSA), has shown future prospect in terms of learning rate parameter determination in waste object detection and classification. Consequently, it is imperative for an EdgeIoT-enabled system to be equipped with Yolov3 for waste object detection and classification, thereby facilitating effective waste collection.

## 1. Introduction

Over the decades, cities have been the main center of business activities, which has resulted in most people preferring to live in urban centers. During business operations, wastes are generated but are not collected frequently, thus leading to a heap of uncollected waste or garbage in cities and urban centers and this poses a major concern in maintaining the quality of health as well as the environment. Cities around the world have an increasing number of inhabitants which directly contributes to the high generation of garbage. An efficient garbage collection process will ensure that wastes are collected in a shorter duration and reduces the negative effects on the environment. Furthermore, by detecting waste quickly, it triggers the necessary intervention from the waste collectors. The efforts toward a clean and neat environment in the face of rapid urbanization is noted as the driving force for the transition to a “smart city” status [1]. Maulana, Widyanto, Pratama and Mutijarsa [2] opine that creating awareness and subsequent changes in the habits of people concerning how waste is managed in cities can help to create a clean environment and possibly enhance quality of life.

Waste management has thus become an issue of concern in every nation and there is a need to develop innovative ways to tackle the malaise. For instance, South Africa has a relatively high rate of waste generation, estimated at 23.21 million tons yearly, but only an estimated 10% of these wastes are recycled [3]. However, rapid urbanization has led to the evolution of innovative methods, such as the “smart city” concept, for managing the complexity of urban waste management systems. Theoretically, smart city uses emerging technologies, such as Artificial Intelligence (AI) and Internet of Things (IoT), to transform the urban environment. This transformation is expected to drive economic growth, improve service delivery, and enhance the quality of life of people. Although the smart city concept has enabled appreciable levels of societal transformation in developed countries, it is yet to be fully explored in developing countries, including South Africa.

The process of solid waste management starts with the collection of waste in containers/refuse bins which have been placed at vantage locations in the public space. Sometimes, the bin is overfilled, thereby creating unhygienic conditions [4,5] which are detrimental to human health and economic activities. Waste objects can be categorized into different types, such as liquid, solid rubbish, organic, recyclable and hazardous wastes. However, these types of waste are less likely to be segregated or sorted by consumers at the point of disposal even when some refuse bins are clearly marked for waste categories. Mostly, people throw all their wastes into a bin without any considerations for recyclability. Thus, it is common to find various waste materials mixed in a bin, and the waste management company manually searches the content of the bin to sort the wastes that comes from homes and/or offices. However, when waste materials are mixed, the separation and processing thereof by the waste management organization becomes a labor-intensive, time consuming and hazardous process [6]. The traditional/manual handling of waste bins by individuals/companies is thus no longer a smart approach to waste management [1].

The use of smart technologies provides the pathway toward sustainable waste management in cities, in the sense that it can help to facilitate monitoring, collection, identification and separation of waste for proper disposal. Although the use of information technology plays a significant role in the application of technology in waste management nowadays, the potential of technologies, such as AI and IoT, is still in its infancy in Africa and the technologies need to be intensified and applied in more strategic areas.

The South African University of Technology (SAUoT) is a public university that champions the use of the smart bin for waste management. This is in line with the institutional commitment toward championing the transition to a smart city status. SAUoT views itself as a real-world, living laboratory for testing various innovations that are expected to facilitate this transition at a city level. Such aspirations are not new as universities across the globe have sought to rely on their tacit knowledge and expertise to drive innovation across multiple scales.

The management of waste is one aspect where SAUoT is seeking to make an impact by leveraging the capabilities of emerging technologies. Currently, the university has waste bins for polystyrene, tins/cans, plastic and paper. However, it lacks the supporting technological devices to assist with waste detection and classification. Hence, the quest to apply technology to solve real-life problems in waste management is one of the motivations for this article because the absence of a smart waste bin system, for instance, has made it difficult for real-time monitoring, detection and classification of waste products.

Consequently, we focus on solving the problem of waste detection by using AI technologies in a manner suggestive of smart waste management. To this end, we provide solutions to the following questions:

(1) What state-of-the-art technology is required to automate waste object detection and classification at the source of the waste collection?
(2) How can a nature-inspired search technique facilitate hyper-parameter tuning for a custom waste object detector?

In this regard, the contribution of this study proposes a custom waste object detector and waste category classification enabled by a nature-inspired hyper-parameter tuning for real-time waste object detection and classification. To the best of our knowledge, using the proposed nature-inspired search method is a novel contribution to custom object detection and classification of waste objects.

This article is organized into the following sections: the review of literature is presented in Section 2. Section 3 focuses on the methodology and materials including the waste images dataset. Section 4 presents the experiment settings and experiment conducted. Section 5 presents the test results, and Section 6 presents the discussion of findings, whereas the conclusion and future work are presented in Section 7**.**

## 2. Review of Literature

Waste management systems generally involve the process of collection, transfer, disposal of waste, sewage and recycling [7]. Sometimes, it is uncertain whether the waste generated at the source is recyclable or not. There are interventions to address this uncertainty, which include the labeling of bins, mostly referred to as “smart waste bin”, which shows the kind of waste to be disposed of therein. A smart waste bin could be considered as an intelligent waste management system, equipped with emerging technologies that decide in real time how waste can be collected and subsequently managed [8]. Intelligence is determined by how quickly and effectively a decision is made by a smart waste bin concerning pre-determined facets [9,10]. A smart waste bin can indicate the volume of waste, the status of the bin (whether damaged or not and its position or location) the use of the bin for the intended purpose and much more.

Information technology plays a vital role in data collection and communication from the smart waste bin to the stakeholder of waste management for quick intervention. Several systems have been proposed for smart waste management; these include the use of IoT-based devices to measure the level of waste and improve the collection of waste [11,12], the use of a Long Range (LoRa) communication system to notify stakeholders on the level of waste in bins [13], detecting when a bin is full to better inform cleaning contractors and litter bin providers to decide on how to increase productivity [14], tagging bins with Radio Frequency Identification (RFID) cards to identify a residence [15], volume-rate waste collection system for food bin using RFID card [15] and, using IoT, microcontroller and ultrasonic sensors to detect the level of waste [4]. The interconnectivity of different IoT devices to enable communication with other devices is crucial in a smart waste management system [16].

Mustafa and Ku Azir [17] proposed a smart waste bin with ultrasonic sensors for measuring garbage levels and it also had a microcontroller to control the system’s operation in determining the status of four different types of trash: domestic waste, paper, glass and plastic through liquid crystal display and the ThingSpeak platform in real time. Sreejith, Ramya and Roja [18] presented a robot smart waste bin equipped with sensors to monitor the level of the bin and when it is filled, it automatically moves it to the waste collection area to dispose of the waste and then it returns the bin to its area. Additionally, a gas sensor is attached to alert people of harmful gas from waste and when rain is detected, the rain detection sensor automatically closes the bin. Similarly, sensors have also been used to measure weight and detect the level of waste in the bin [19]. In some instances, sensors have been used to send data on a volume of trash to an online server for processing [6]. Vu and Kaddoum [20] applied sensors to detect, measure and transfer data on the volume of waste over the internet. Joshi, Reddy, Reddy, Agarwal, Agarwal, Bagga and Bhargava [21] modeled a smart waste bin based on the Stack Based Front End method that integrates with the IoT Wireless Sensor Network connected to the cloud computing environment.

Maulana, Widyanto, Pratama and Mutijarsa [2] modeled a waste management system with three components: the trash bin with a sensor, a communication system to inform stakeholders on the status of waste, and a waste scheduling control. Although the system uses sensors to detect different types of waste and to send and display the status of waste to the waste management station, it is unable to identify whether the different types of waste are recyclable or not.

The idea of source reduction and recycling of waste is one of the effective ways to address the challenge of waste disposal. IoT-based serverless architecture attached to bins has been proposed to keep track of waste from the source and identify violation points when waste is not placed in the correct bin [8]. When a smart waste management system is equipped with IoT-enabled devices, it can help to identify the kind of waste material [8]. Some interventions on waste material identification from the source of generation involve the use of connected devices [22] to detect and capture images on any kind of trash. With Artificial Intelligence (AI) and Machine Learning (ML) algorithms, captured images of waste at the source of generation can be processed by extracting the unique traits from the image to classify the nature of materials according to their recyclability or otherwise [23].

Because a significant amount of waste is generated daily in communities, the deployment of an intelligent system for managing such waste becomes imperative. This brings to the fore the role of AI as one of the emerging technologies that has been deployed in waste management systems. AI connotes the ability of a computer to perform tasks, such as reasoning and learning, that human intelligence can perform [24]. It involves the design of intelligent agents that can check their environment, reason, analyze and take the appropriate action based on the available data. By designing intelligent agents, machines can learn, adapt and move closer to artificial intelligence [25]. One of the devices that uses artificial intelligence is an IoT-enabled device or an edge computing device. By attaching IoT-enabled devices to a waste bin, we can deploy a smart bin for the waste management system. Hulyalkar, Deshpande, Makode and Kajale [23] proposed a machine learning model based on convolutional neural network (CNN or ConvNet) for automatic waste segregation. CNN is a deep learning model that can be used to perform a classification task [26] directly on objects. The proposed CNN model extracted unique traits of the object in an image and then classified the same into predetermined classes: plastic, metal, paper and glass. Machine learning algorithms, such as Artificial Neural Networks (ANNs), Support Vector Machine (SVM), and Recurrent Neural Network (RNN) were found not to outperform the proposed CNN model as the chances of obtaining the exact image are high in the CNN model even if the number of image dataset is increased, as stated by Vu and Kaddoum [20]. CNN improves the image search when a large number of images are involved and this enhances the building of a very high-level feature for image detection [27]. Sunny, Dipta, Hossain, Faruque and Hossain [28] proposed a smart dustbin—Automated Teller Dustbin (ATD)—that uses CNN to detect and recognize waste and determines the recycle value in direct exchange for money. The Dynamic Time Warping (DTW) and Gabor Wavelet (GW) have also been used to extract features of waste images and trained Multi-Layer Perceptron (MLP) classifier was employed to estimate the amount of waste in a bin [29].

In [30], waste recycling in smart cities was proposed where a deep reinforcement learning based model was used to detect and classify waste using deep learning techniques. The proposed system was anchored on a two-stage process called masked regional convolutional neural network (Mask-RCNN) and Deep Reinforcement Learning (DRL) for waste detection and classification. The Mask-RCNN model used the DenseNet model as its baseline model whereas the classifier adopted is a Deep Q-Learning Network (DQLN). In improving the efficiency of the DenseNet model, a nature-inspired dragonfly algorithm based hyper-parameter optimizer was developed. The performance of the proposed model was evaluated using simulations on a benchmark dataset and the experimental results obtained indicate the best accuracy of 0.993 for waste detection and classification.

The hybrid transfer learning for classification and faster R-CNN was used to obtain region proposals for object detection [31]. This hybrid approach used various wastes in a collaged image classified into six categories, that is glass, plastic, paper, trash, metal and cardboard. The TrashNet dataset consisting of 400 waste images in each of the labelled categories was used for experimentation and divided into training, validation and testing set. With varied learning rates and while using precision, recall and F1-score as evaluation metrics, the best results of 0.97, 0.99 and 0.98 for precision, recall and F1-score, respectively, were achieved for the cardboard category.

A smart dustbin prototype was designed by [32] such that the lid of the dustbin is opened when human hand and waste is detected while the volume of waste in the bin is sent as notification in the form of Light Emitting Diode (LED). The major components of the prototype include Arduino, NODEMCU, Servo Motor and Ultrasonic Sensors whereas the Blynk application is the software component that receives notification. The bin is considered useful in the smart waste management system where the solid waste workers can clean or empty the bin depending on the notification received rather than unnecessary physical visits or waiting for a call from households to inform and request for garbage trucks to evacuate waste.

Gupta, Shree, Hiremath and Rajendran [6] opines that an effective and proper waste management system does not end with collection and disposal but also recycling, which is so useful because of society’s worrisome dependence on finite raw materials. However, the reduction in human involvement in the manual search of waste bins to identify non-recyclable materials poses a challenge. By leveraging IoT or edge computing devices and artificial intelligence models, the manual search for waste and its separation can be replaced with a real-time waste identification system.

### Traditional Waste Management Framework

The traditional waste management framework consists of three layers: the physical infrastructure layer, the hardware layer, and the analytics layer for advanced data processing [33]. The physical infrastructure layer is responsible for the physical elements used in waste management whereas the hardware layer helps to control the physical infrastructure in terms of the tracking movement of the physical elements, such as the trucks, and not the waste bin [34]. On the other hand, the analytics layer consists of the software to manage the general operations of the waste management. The challenge with the traditional framework is that it is unable to automatically identify or capture images of trash for further processing into the categories of waste materials in the bin. Considering the case of SAUoT, where the waste bins are not equipped with AI and IoT-enabled devices, the waste management system follows the traditional waste management framework. Thus, the traditional waste management framework is not robust to address the current challenges of waste management, particularly as it pertains to resolving the challenges associated with manual identification, classification and sorting of waste materials. This will contribute to the circular economy mandate of closing the material loop [33].

## 3. Methodology and Materials

The state-of-the-art YOLO algorithm that uses CNN-based method was employed and combined with the nature-inspired algorithm for waste object detection and classification. In this study, we firstly collected data on waste images that were labeled with ground truth boxes for training. The following section discusses the method in detail.

### 3.1. Convolutional Neural Network

The YOLO algorithm employs Convolutional Neural Networks (CNN) to detect objects in real time. The CNN based model for object detection can be categorized into region proposal (R-CNN) based and regression/classification based [35]. The R-CNN based model leads to high accuracy, but it is unable to achieve real-time speed, whereas the regression/classification-based model has an optimal computational cost. In tackling the object detection problem, the accuracy of detection, how quickly an object is detected, and the computational cost are issues of concern in deploying systems that require real-time performance. An object detector consists of two parts: a backbone, which is pre-trained on ImageNet; and “a head”, which predicts a class and bounding boxes (BBoxes) of objects [36]. The accuracy of predictions is calculated based on Average Precision [37] and a lower loss value of an image shows better performance. An example of a regression based CNN object detection model is You-Only-Look-Once (YOLO) [38]. YOLO uses a deep learning algorithm to detect objects in real time and it is a single-stage, real-time object detection model [39]. The different versions of YOLO are Yolo version 2 (Yolov2), Yolo version 3 (Yolov3) and many more. The concept of YOLO can be summarized as follows:

(i) The unification of separate common components in object detection into one single neural network.
(ii) Use of features of an entire image to predict bounding boxes.
(iii) Concurrent prediction of all bounding boxes for each class.

There are techniques to perform regression on center point coordinates and, height and width of the bounding box and the mean square error (MSE) is one of such techniques. However, to directly estimate the coordinate values of each point of the Bbox, it is necessary to consider the points as independent variables. One of the techniques to achieve this is by Intersection Over Union (IoU) loss [40]. The IoU loss considers the coverage of the predicted Bbox area and ground truth Bbox area to ensure the calculation of the coordinates of the Bbox [36]. Another technique is GioU loss [41], which predicts the shape and orientation of objects ©n addition to the coverage area. The DIoU loss [42] technique considers the distance of the center of an object whereas cIoU loss [42] considers overlapping area, the distance between center points and the aspect ratio, thereby ensuring accuracy and speed of convergence on the Bbox. Furthermore, the anchor-based technique is applied to estimate the corresponding offset on coordinates [36] whereas the mean Average Precision (mAP) is a technique used to measure the accuracy of the object detector.

**Figure 1:**
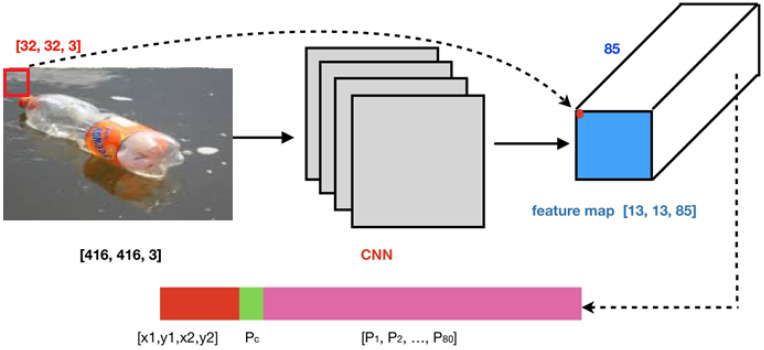
Basic structure of ROI area mapped on the CNN network.

Figure 1 depicts a Region of Interest (ROI) on the upper left corner of the input image being mapped by CNN to a feature map, which becomes smaller or is only one point with 85 channels. So, the dimension of ROI changes from the original [32, 32, 3] to the 85-dimension. Any grid on the input then outputs a bounding box ([x1, y1, x2, y2]), confidence (Pc), and class probability map ([P_1_, P_2_, …, P_80_]).

**Figure 2:**
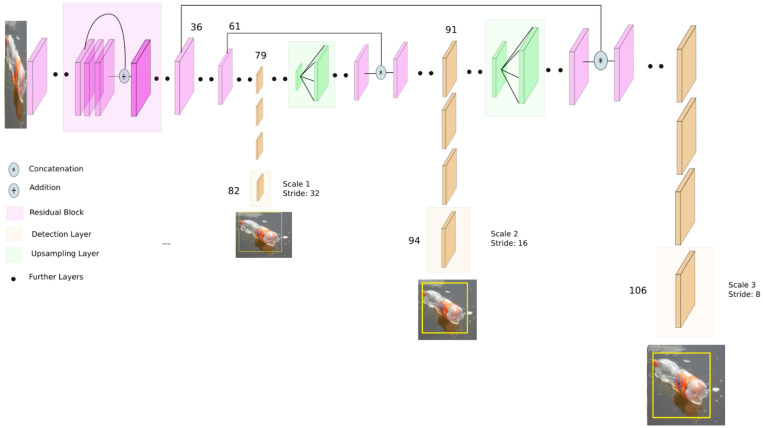
Structure of the YOLO deep learning network.

Figure 1 shows the structure of ROI area mapped on the CNN network. Generally, the structure of the YOLO deep learning network, as shown in Figure 2, is such that, for any input image, YOLO detects in three different scales to accommodate various objects sizes by using strides of 32, 16 and 8. This indicates that if an image of size 416 × 416 is input, YOLOv3 detects on the scale of 13 × 13 × 255, 26 × 26 × 255 and 52 × 52 × 255 after entering the Darknet-53 network. After that, YOLOv3 picks the feature map from the layer 79 and then applies one convolutional layer before upsampling it by a factor of 2 to form a size of 26 × 26. The upsampled feature map is concatenated with the feature map from the layer 61. Afterwards, the concatenated feature map is subjected to a more convolutional layers until the second detection scale is performed at layer 94. The second prediction scale produces a 3-D of size 26 × 26 × 255.

### Steps for Object Detection Using YOLO

The detection of objection using the YOLO model involves the following steps:

- Start by dividing an input image into the N × N grid cell.
- If the object falls in the center of the grid cell, then it is responsible for detecting that object. Then, each grid cell predicts the bounding boxes and confidence scores for the predicted boxes.
- Each bounding box consists of five predictions: *x, y, w, h* and confidence. The *(x, y*) coordinates represent the center of the box relative to the bounds of the grid cell. The width (w) and height (h) are predicted relative to the whole image.
- Confidence is the probability of an object existing in each bounding box, expressed as:
  $$Pr\;\left(Object\right)\;\times \;IOUground\;truth$$
  where *IoU* is Intersection Over Union. Intersection is the area that overlaps between the predicted bounding box and ground truth, whereas the union is the total area between predicted and *ground truth*.
- By using the confidence scores, the certainty of an object being in the box as well as the precision of the model’s prediction is guaranteed. However, if there are no objects, then the confidence scores are zero. Each cell also predicts the class conditional probabilities of an object *Pr(Class|Object).* The class-specific confidence scores for each box [32] are expressed as:
  $$Pr(Classi|Object)\times Pr\left(Object\right)\times \;IOUground\;truth=Pr\left(Classi\right)\times \;IOUground\;truth_{\;}$$
- To optimize the confidence scores, the loss function as expressed by [37] is used to correct the error in the center and the bounding box of each prediction.
- The model’s accuracy of prediction is calculated in Average Precision (AP%).

### 3.2. CNN Model Implementation Platform

This indicates how the CNN platform is implemented in this study. The AI-based application program enables the detection of labelled images in the advanced analytics layer and the Darkflow helps build the TensorFlow network from files and the pre-trained weights. Darkflow has all the tools that are necessary for training and testing experiments, except the pre-trained “*weight*” file that needs to be obtained from the YOLOv3 settings (https://pjreddie.com/darknet/yolo/ (accessed on: 23 October 2020)). After that, a Python script was created with information about the location of the dataset consisting of the waste objects’ images in this regard, the location of the labels or classes or categories and the used network architecture. The Python script was executed to start training the Yolo model for the custom object detection [35].

### 3.3. Edge Computing Enabled IoT (EdgeIoT) Framework

**Figure 3:**
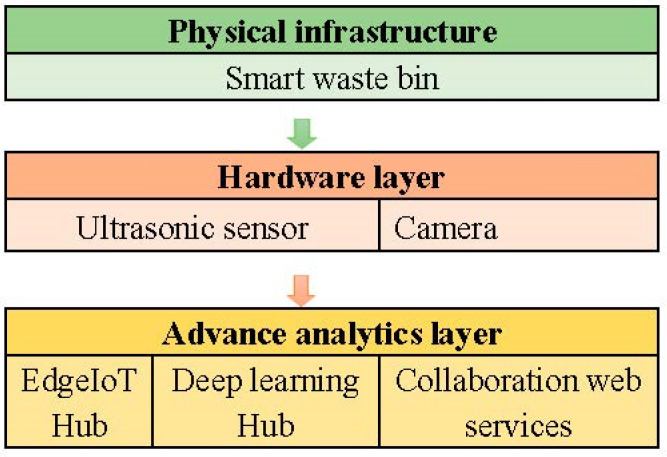
EdgeIoT smart waste bin framework.

The smart waste bin collects different categories of waste, such as polystyrene, tins/cans, plastic and paper and then classifies the trash according to the different categories. The fundamental problem is that often waste materials are mixed, thereby necessitating manual searches through the content of the bin to sort the waste into different categories. Waste management systems can rely on technology to provide a collaborative human–computer platform for effective waste management. The smart waste bin captures images of the different categories of waste and classifies the waste into separate categories when waste is detected. The framework consists of three layers: the physical infrastructure, the hardware layer and the advance analytics layer, as shown in Figure 3.

The physical infrastructure consists of the smart waste bin where each bin is assigned a serial number for easy identification. Each of these bins is equipped with an EdgeIoT device that connects the hardware components [8].

The hardware layer consists of an ultrasonic sensor and a camera. The ultrasonic sensor measures the level of a dustbin while the camera captures the images of the waste. The captured images are then sent to the advanced analytics layer via an EdgeIoT hub.

The advanced analytics layer consists of edge computing IoT devices, deep learning models for object detection and classification, and collaboration web services for integration with an online system for awareness creation on the categories of waste. The framework process images either on a cloud-based parallel Graphics Processing Unit (GPU) platform without additional hardware investment or reside locally on the waste bin. Finding the best image detection model is often a challenging task especially when it requires the transitioning from a manual system to an automated system.

Our approach employs the state-of-the-art YOLO algorithm, which has the CNN model [43] as the underlining framework, for waste object detection. YOLO was chosen because of its ability to group common features of an object into a single neural network and make concurrent prediction, thus making it suitable for this research. It is imperative to have an algorithm that performs real-time object detection at a minimal computation cost when developed on an edge computing enabled IoT device. Such devices work with maximum accuracy only on the image containing a single object, which is a major shortcoming of these devices, such as Raspberry Pi and Arduino, and their respective algorithms [44]. The EdgeIoT device contains the image detection algorithm and the EdgeIoT hub is considered as the application center.

### 3.4. Design Prototype of Smart Waste Bin

**Figure 4:**
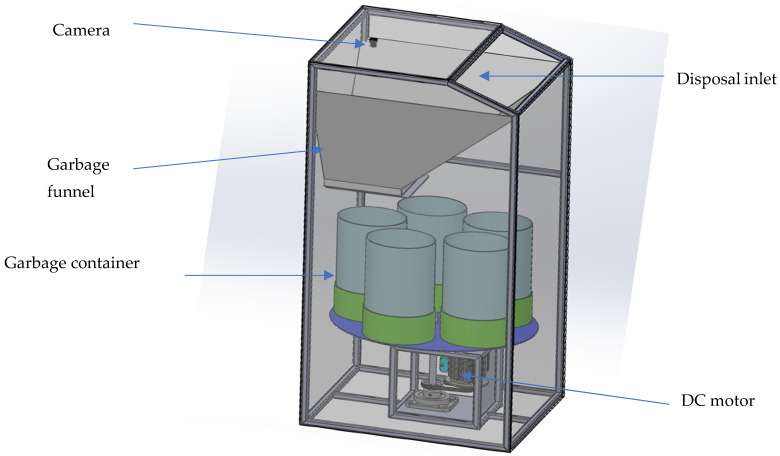
Design prototype of smart waste bin.

The design prototype of the smart waste bin is shown in Figure 4.

The design smart waste bin prototype consists of a servo motor, garbage bin, garbage funnel, disposal inlet and camera. The garbage funnel is equipped with the AI software and edge device component. The garbage funnel temporarily holds the garbage for capturing by the camera in bright lighting condition and processing by the embedded edge device. The disposal inlet is the opening of the smart bin. The garbage container holds the classified waste whereas the Direct Current (DC) motor spins the garbage container to the valve of the garbage funnel.

**Figure 5:**
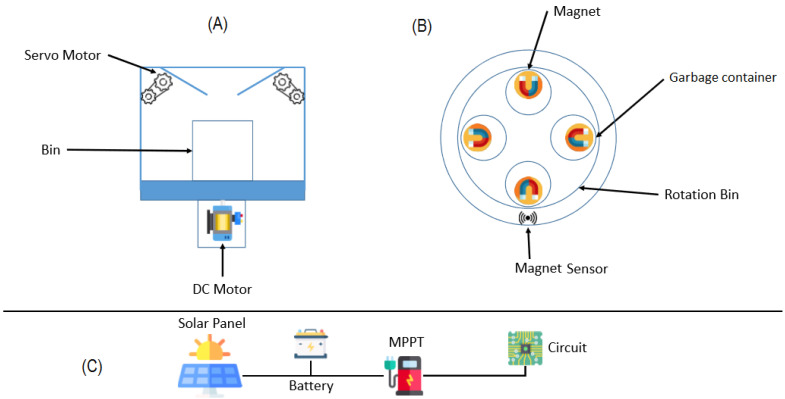
Schematic representation of smart waste bin.

Figure 5 below shows the proposed schematic representation of the smart waste bin. This schema shows all the components that make up the smart waste bin, where (Figure 5A depict the design prototype of smart waste bin in the Figure 4 above, Figure 5B shows the magnet sensor, magnet, smart waste bin and rotation bin and Figure 5C shows the solar panel, battery unit, Maximum Power Point Tracker (MPPT) and circuit.

In Figure 5A, the servo motor allows control at the garbage funnel valve on the smart waste bin. In Figure 5B, the magnet sensor detects the position of the garbage container in the smart waste bin. In Figure 5C the solar panel, battery unit and Maximum Power Point Tracker (MPPT) combined provide power to the circuit of the smart waste bin, which is the proposed embedded device. The proposed embedded device (that is, LattePanda alpha 864) equipped with a camera acts as the advance analytics layer that resides locally on the waste bin, which is ideal for the edgeIoT-based system and represents the central processing system to control the smart waste bin. The programmable circuit and Integrated Development Environment (IDE) ensured the upload of the proposed waste detection and classification model on the physical board. LattePanda is a high-performance minicomputer integrated with Arduino with low power consumption capability. The hardware specification of the LattePanda alpha 864 is presented in Table 1. The specification of the camera is the 5 MP USB Camera module, which supports OTG, Auto-Focus, Automatic Low Light Correction capability and Plug and Play.

### 3.5. KSA-Based Nature-Inspired Search

**Figure 6:**
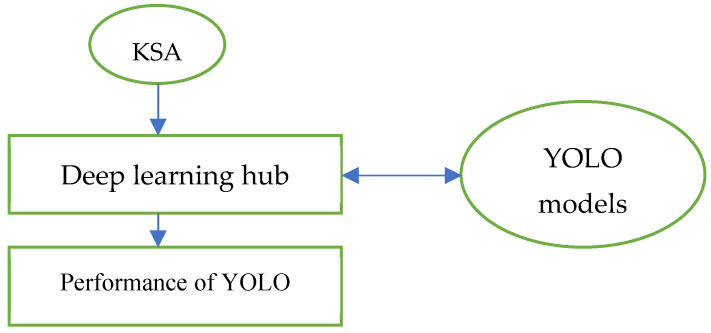
Model for KSA and deep learning hub.

The approach to fine tune the default learning rate of the YOLO model is based on the nature-inspired behavior of a bird called Kestrel. Kestrel belongs to the falcon family; it hovers and perches to hunt its prey. Kestrels are capable of learning by hovering in changing airstreams, maintaining a fixed forward-looking position with their eyes on the prey and using random bobbing of the head to learn the shortest distance to its prey. Additionally, Kestrels are naturally endowed with ultraviolet sensitive eyesight that can trail urine and feces reflection. The Kestrel-based search algorithm (KSA) is governed by three basic rules: improve, reduce and check rules, which are detailed in [26,45,46]. The Improve Rule (IR) and Reduce Rule (RR) are employed to facilitate the hyper-parameter tuning for a custom waste object detector of the deep learning hub. Figure 6 shows the model for KSA and the deep learning Hub.

Figure 5 consists of the KSA, deep learning hub, YOLO models and the performance of the YOLO model. The KSA algorithm outputs its optimal hyper-parameter to the deep learning hub to detect the waste’s image. The deep learning hub consists of YOLO models, as discussed in Section 3.1 of this article.

The improve and reduce rules of the KSA are expressed as follows:

***A.***  ***Improve rule expression***

The detailed expression of the improve rule is indicated in [26,45,46] and the simplified rule can be expressed as:

(1) $$x_{i+1}^{k}=\vec{x}\left(t+1\right)+\beta_{o}e^{-\gamma r^{2}}\left(x_{j}-x_{i}\right)+f_{t+1}^{k}$$

where $x_{i+1}^{k}$ represents the current best position of a Kestrel bird. Thus, candidate solution $\vec{x}\left(t+1\right)$ is the previous Kestrel’s position obtained from the random encircling formulation [47], $\beta_{o}e^{-\gamma r^{2}}$ represents the attractiveness that relates to the light reflection where the variable $\beta_{o}$ is the initial attractiveness, $x_{j}$ represents a Kestrel with a better position, $x_{i}$ also represents a previous position of a kestrel, *r* represents sight distance $s\left(x_{i},\;x_{c}\right)$ measurements which are expressed based on the Minkowski distance, γ is “light intensity of variation” between [0, 1], $x_{j}$ represents kestroid in better position and $f_{t+1}^{k}$ is the “frequency of bobbing” to detect its prey within sight measurement expressed as:

(2) $$f_{t+1}^{k}=f_{min}+\left(f_{max}-f_{min}\right)\times \alpha$$

where $\alpha \;∊\;\left[0,\;1\right]$ is a random number between 0 and 1 that controls the bobbing frequency within a visual range. In addition, *fmax* and *fmin* are the maximum and minimum frequency set between 1 and 0.

***B.***  ***Reduce rule***

This rule depicts the unstable nature of Kestrel as the energy it exerts in searching gradually reduces. Hence, the unstable nature of energy exerted can be expressed as: if there is *N* unstable energy exerted, then the rate of energy exerted decay with time t is expressed as:

(3) $$\frac{dN}{dt}=-\gamma N$$

subsequently re-simplified as:

(4) $$\gamma_{t}=\gamma_{o}e^{-\varphi t}$$

The decay constant φ is how long energy source can decay which is mathematically expressed in Equation (5) as:

(5) $$\varphi =\frac{\ln0.5}{-t_{\frac{1}{2}}}$$

and $t_{\frac{1}{2}}$ is the period of the half-life. A decay constant of more than 1 indicates the trail is fresh, otherwise it is old; this is re-expressed in Equation (6) as:

(6) $$if\;\varphi \;\to \;\left\{\begin{array}{c} \;\varphi >1,\;trail\;is\;new\;\\ 0,\;otherwise\; \end{array}\right.$$

#### Algorithm to implement the KSA-based rules

The KSA-based algorithm can be summarized into two parts: Algorithms 1 and 2, as follows:

*Start*: Set Flight zmax = 0.8, Perched zmin = 0.2, attractiveness $\beta_{o}=1$.


**Algorithm 1: Improve Rule (IR)**

*Step 1: Compute*

$\vec{x}\left(t+1\right)$


*Step 2: Compute*

$\beta_{o}e^{-\gamma r^{2}}$

*Step 3: Find*γ at time *t* from the reduce component
*Step 4: Compute*

$f_{t+1}^{k}$


*Step 5: Compute*
**best position**

$x_{i+1}^{k}=\vec{x}\left(t+1\right)+\beta_{o}e^{-\gamma r^{2}}\left(x_{j}-x_{i}\right)+f_{t+1}^{k}$




**Algorithm 2: Reduce Rule (RR)**

*Step 1: Compute*

$\gamma_{t}=\gamma_{o}e^{-\varphi t}$


*Step 2: Compute*

$\varphi =\frac{\ln0.5}{-t_{\frac{1}{2}}}$



$if\;\varphi \;\to \;\left\{\begin{array}{c} \;\varphi >1,\;trail\;is\;new\;\\ 0,\;otherwise\; \end{array}\right.$



*End*: Output the optimal learning rate parameter.

Algorithm 1 represents the Improve Rule (IR), which consists of five key steps, expressed using the mathematical equations in Section 3.5. The algorithm process starts with the initial parameters, namely flight, perch and attractiveness, and then performs the computation to find the best position.

Similarly, in Algorithm 2, the Reduce Rule (RR) consists of two steps, which are expressed using the mathematical Equations (4)–(6). In Algorithm 2, the output of step 1 is fed into step 3 of Algorithm 1.

### 3.6. Waste Image Dataset

**Figure 7:**
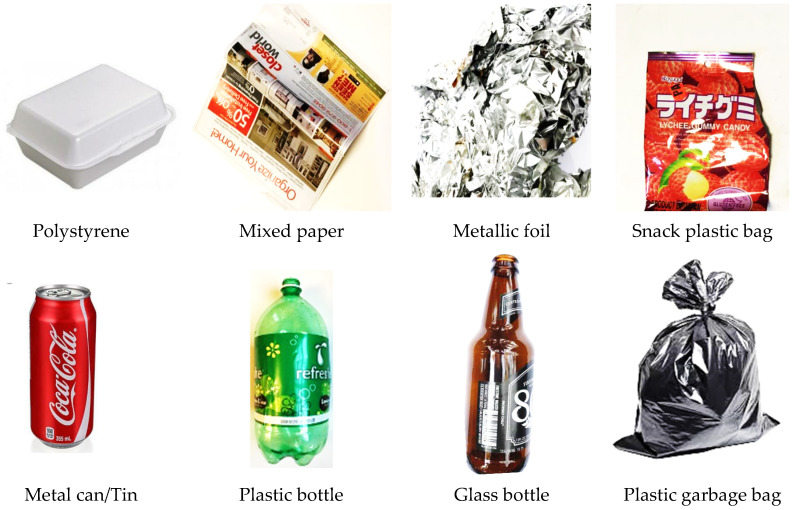
Samples of waste image datasets.

In this article, the custom dataset is a dataset that we created from waste images obtained online (https://arxiv.org/abs/1405.0312 (accessed on 10 January 2021). The custom dataset images were labeled and there are eight classes or categories of waste items, as presented in Table 2 and Figure 7. The input images consisting of 3171 waste images are in the “.jpg” format and the size is reduced to ensure a lighter image is passed through the network for fast learning and minimum computation cost while sustaining the same amount of information during training [48]. Eighty percent of waste image data was used for training whereas 20% was for testing, as it is imperative to train the custom model with a large amount of data.

## 4. Experimental Settings

The YOLO configuration file, YOLO weights and backbone were used for the experiment conducted. An object file in the format of “.*data*” and “.*names*” was created to support the YOLO configuration file, where “.*data*” has information on train, test, backup and number of classes and “.*names*” consists of the list of classes of waste whereas the YOLO configuration file in the format of “.*cfg*” consists of the network architecture. In [36], it was suggested that the backbone improves object classification and detection in datasets, such as the Microsoft Common Object in Context (MS COCO) dataset that we used. In this study, the backbone for each YOLO model of our custom dataset is presented in Table 3.

The backbones are pre-trained convolutional weights for object detection. The primary difference between YOLOv4-tiny and YOLOv4 is the number of layers, which is two and three fully connected layers, respectively. In addition, the number of layers in YOLOv3-tiny and YOLOv3 are two and three, respectively, and there are fewer anchor boxes for prediction in YOLOv3-tiny and YOLOv4-tiny. This study considered the chances of limited computing resources of edge computing devices, hence the consideration of YOLO with two layers. It is significant and necessary to find the best suitable model for a real-life application that depends on the accuracy and/or speed of prediction.

### Hyper-Parameter Settings of Network Structure

The default hyper-parameters for the YOLO model are as follows: the max_batches is 16,000; the batch size and the mini-batch size are 64 and 32, respectively; the learning rate 0.001; step is 12,800 and 14,400, which represents 80% of the max_batches and 90% of max_batches, respectively; the momentum and weight decay are respectively set as 0.9 and 0.0005 whereas the default activation function for the YOLO model used is Leaky Rectified Linear Unit (ReLU) activation function. In setting the environment for the iteration, each class of waste is executed in 2000 iteration; thus, considering the number of the classes of waste (8) results in a total of 16,000 iterations. The input resolution of 416 × 416 was used during the experiment for each YOLO model except Yolov4, which was 608 × 608. The hyper-parameters for the YOLO models are presented in Table 4.

The YOLO configuration file, supporting object files and respective backbones, was trained on the Google cloud GPU to obtain the final “.*weights*”, which represents the YOLO custom detector model that is used for image detection. Finally, we used the steps for object detection using YOLO to detect and predict the different classes of the waste images on a Central Processing Unit (CPU).

## 5. Presentation of Test Results

We used the YOLO configuration file, supporting object “.*data*” file and the YOLO custom detector model for object detection. During testing, the YOLO configuration file was set to the testing mode, which means setting both the parameter for batch and sub-divisions to 1. In this article, we used the YOLO model to detect waste items and a different test dataset to test the model because we present the results of the accuracy of detection in Table 5 using confidence value of objects between 0 to 1, which represents 0% to 100%. There were 22 different images tested, and a lower loss value represents the higher performance of detection.

**Figure 8:**
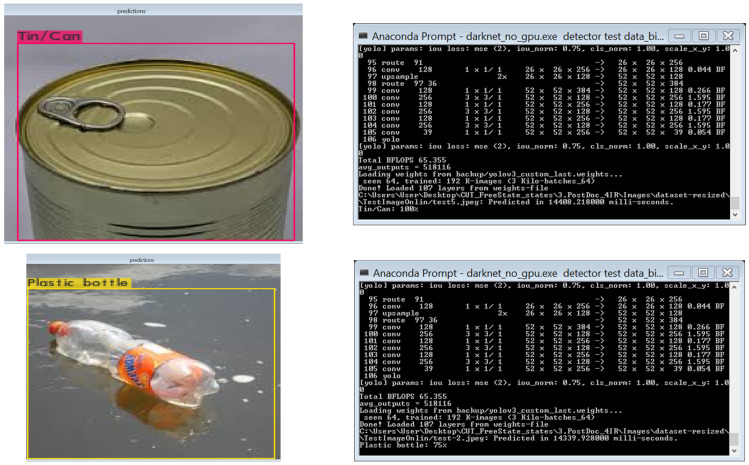

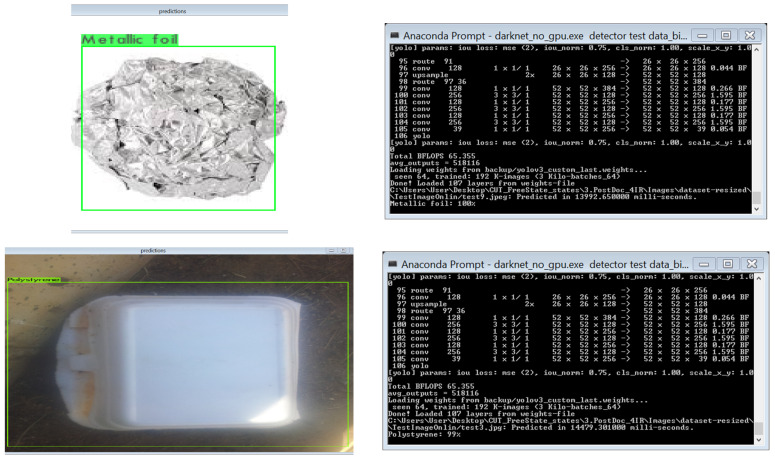
Samples of waste image tested using Yolov3 on “Anaconda Prompt”.

Figure 8 shows the samples of waste images tested using “Anaconda Prompt” on the Yolov3. Testing was performed using the “darknet no gpu” executor file, which also displays the accuracy of detection of waste objects. The “darknet no gpu” executor file was used because it supports CPU based computers.

Table 5 shows the results of the performance of the YOLO models. The performance results of the classes of a waste dataset for metal cans/tins are 100%, 99%, 57% and 72% for Yolov3, Yolov3-tiny, Yolov4 and Yolov4-tiny, respectively.

Table 6 shows the performance results of each trained version of the YOLO model where the accuracy of detection of each trained version of YOLO model is measured in Average Precision (AP%) and mAP.

The mAP (%) for each trained version of YOLO models is Yolov3 (80%), Yolov4-tiny (74%), Yolov3-tiny (57%) and Yolov4 (41%). This result of mAP (%) indicates that the Yolov3 model produced the best performance results (80%). In Table 7, the Billion Floating Point Operation per Second (BFLOPS) indicates the number of operations that were performed in seconds on a single waste image with a resolution of 416 × 416 for Yolov3, Yolov3-tiny and Yolov4-tiny and 608 × 608 for Yolov4.

Table 7 shows the BFLOPS and the number of layers that were loaded from the trained files during the testing. It is observed that Yolov3-tiny, Yolov4-tiny, Yolov3 and Yolov4 have BFLOPS of 5.459, 6.798, 65.355 and 127.34, respectively. Generally, this means Yolov3-tiny processes a single waste image with a resolution of 416 × 416 faster as compared with Yolov4-tiny, Yolov3 and Yolov4. The reason is because of the number of layers that were loaded from the trained file. Again, Yolov3-tiny, Yolov4-tiny, Yolov3 and Yolov4 have 24, 38, 107 and 162 layers, respectively, loaded from the trained file.

Table 8 shows the speed of prediction (measured in milli-seconds) on test images. The speed of prediction indicates how fast the model predicts the image.

Table 8 shows the results of the speed of prediction for each waste image that was tested. The speed to predict a newspaper is 14,864.519, 1713.719, 38,687.950 and 2387.265 for Yolov3, Yolov3-tiny, Yolov4 and Yolov4-tiny, respectively. In addition, the speed to predict metal cans/tins is 14,408.218, 1787.309, 37,195.259 and 2011.539 for Yolov3, Yolov3-tiny, Yolov4 and Yolov4-tiny, respectively. Again, the average speeds of prediction (in seconds) are 14.51 (Yolov3), 1.72 (Yolov3-tiny), 38.95 (Yolov4) and 2.20 (Yolov4). A further experiment result on the speed of prediction is presented in Table 9.

Part of the contribution of this article is the use of the nature-inspired algorithm to find the learning rate. Table 10 shows the learning rate generated by the KSA for the experiments conducted for five (5) iterations.

Table 10 shows the optimal value as 0.0007 in iteration #4. This was used as the learning rate (0.0007) for YOLO models to identify the best performing YOLO model. The performance results are presented in Table 11.

**Figure 9:**
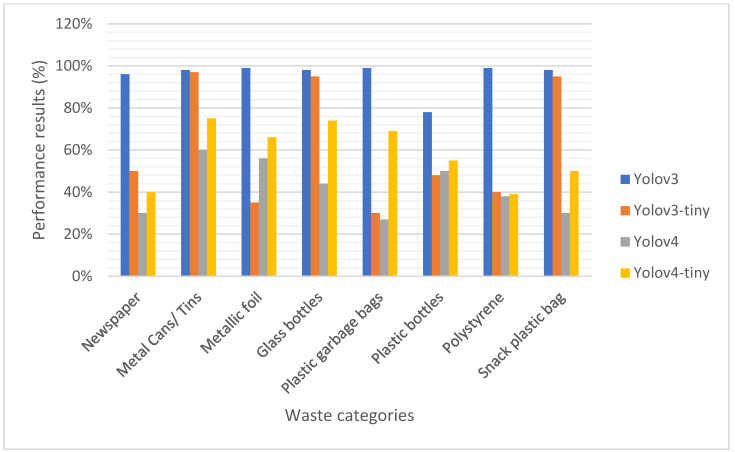
Performance results of KSA-based YOLO model.

The results in Table 11 show that by using KSA for parameter tuning, Yolov3 is the best with an Average Precision of 96%. Figure 9 shows the performance results graph of the KSA-based YOLO model.

## 6. Discussion of Findings

The results in Table 5 indicate that the Yolov3 detected metal cans/tins image with 100% accuracy as compared with the Yolov3-tiny (99%), Yolov4 (57%) and Yolov4-tiny (72%). In addition, the accuracies of the detected newspaper images are 97%, 0%, 28%, and 35% for Yolov3, Yolov3-tiny, Yolov4 and Yolov4-tiny, respectively. The zero percent (0%) showed that the respective Yolo model could not detect the waste images; hence, there was no predicted value. In this regard, for the Yolov3-tiny model, newspaper, plastic garbage bag and polystyrene wastes were not detected. Similarly, the Yolov4 model was unable to detect the plastic garbage bag. The Yolov3-tiny detected the plastic snack bag with 100% accuracy as compared with Yolov3 (99%), Yolov4-tiny (54%) and Yolov4 (32%). However, the performance results of Yolov3-tiny were not the best across the other waste objects.

The results in Table 7 indicate that Yolov3, Yolov3-tiny, Yolov4 and Yolov4-tiny spent approximately 14.41 s, 1.78 s, 37.19s and 2.0 s, respectively, to detect metal cans/tins. This suggests that the Yolov3 spent 14,408.218 milliseconds or 14.41 seconds to predict the metal cans/tins of resolution 416 × 416. Whereas Yolov3-tiny spent 1787.309 milliseconds (or 1.78 s), Yolov4 with a resolution of 608 × 608 spent 37,195.259 milliseconds (or 37.18 s) and Yolov4-tiny was 2011.539 milliseconds (or 2.0 s). In detecting the plastic snack bag, the Yolov3-tiny spent 1832.831 milliseconds (1.8 s) with 100% accuracy, Yolov3 spent 13.8 s with 99% accuracy, Yolov4-tiny spent 2.3 s with54% accuracy and Yolov4 spent 37.9 s with 32% accuracy. Thus, Yolov3-tiny is faster at prediction but less accurate, whereas Yolov3 is accurate but slow in prediction. The test results on accuracy of prediction for each Yolo model suggest that Yolov3 is best at detection. The number of layers loaded from the trained file during testing was 107, which impacts the speed of detection. Though it may be expected that Yolov4 with 162 layers and 127.34 BFLOPS could produce accurate prediction results, it did not. Shah, Panigrahi, Patel and Tiwari [49] indicated that the slow processing time per frame in a model such as YOLO is due to the rationale behind the design of the convolution architecture, which is the number of layers. Wu, Chen, Gao and Li [50] indicated that the Yolo algorithm is much faster than other detection algorithms, such as Faster R-CNN, when applied as a detection algorithm for airports. However, the Yolo algorithm was said to be less accurate. In [51], Yolov3 with darknet-53 as the backbone achieved the best performance among the comparative models when applied in a real-time pattern recognition of 331 GPR images. Redmon and Farhadi [52] indicated that the difference between the Darknet-53 and Darknet-19 backbone is usually the size. In addition, the speed and accuracy with the “bigger network” are slower but more accurate [52]. Alderliesten [53] indicated that the accuracy of Yolov3 increases “dramatically”; however, it is constrained with the speed of prediction. The constraint with speed was attributed to the number of layers which was 106 layers fully convolutional [53]. Comparing our results on accuracy with Shah, Panigrahi, Patel and Tiwari [49] on the metal can/tin class, our Yolov3 had an AP of 96.8% whereas that of Shah, Panigrahi, Patel and Tiwari [49] was 98.609%. Again, with glass bottles, our Yolov3 had an accuracy of 97.6% AP, whereas that of Shah, Panigrahi, Patel and Tiwari [49] was 72.987%. Furthermore, in this article, the accuracy of predicting “glass bottle” by Yolov3 is 97.6% AP, whereas in [54] the accuracy was 94%. In some instances, our Yolov3 with darknet53.conv.74 backbone gave more highly accurate results than that of Shah, Panigrahi, Patel and Tiwari [49], which used darket53 backbone. The findings suggest that Yolov3 may be best at detecting waste images, which was also confirmed by Kumar, Yadav, Gupta, Verma, Ansari and Ahn [54]. It was indicated by [54] that the performance of “any deep learning model is highly influenced by the size of the dataset”. A study by [54] used 5150 waste images that were trained on Yolov3 and Yolov3-tiny, which revealed that the performance of Yolov3 was better than Yolov3-tiny. This was confirmed in this experiment where Yolov3 was 80% mean AP and Yolov3-tiny was 57% mean AP. In addition, in [54] it was revealed that the Yolov3-tiny processes waste images faster (1.72 s) than Yolov3 (14.51 s), as evident in this study’s experiment.

Among the nature inspired algorithm used in image detection is the dragonfly algorithm for the hyper-parameter optimizer, which enhances the detection [30]. Similarly, KSA when used to determine hyper-parameter, can also guarantee accuracy, as suggested by our experiment.

Though a smart dustbin prototype has been proposed by [32] with underpinning Blynk application software, our study also proposes a smart waste object detection and classification algorithm based on the Yolo model. Considering the case of SAUoT, which operates a traditional waste management system, the implication of the results of this experiment is that it is imperative to implement a system that can accurately classify waste. Therefore, the Yolov3 model can be adopted to transition the traditional waste management system of SAUoT into an automated system. Hence, this study proposes the EdgeIoT model, which uses Yolov3 with darknet53.conv.74 backbone and the Kestrel-based nature-inspired algorithm to fine tune the learning rate of a network structure.

## 7. Conclusions and Future Work

In this article, we propose a framework to enable the transition from a manual waste bin system to an IoT-based smart bin system for waste management on SAUoT’s campus. The YOLO model was applied as the deep learning convolutional neural network model to detect the custom waste image dataset. This model was chosen because of the accuracy and speed of prediction. In addition, the underlying algorithmic structure for the operation of the smart waste bin was implemented in Python. The Google cloud GPU platform was utilized to train the custom YOLO model object detector for the eight classes of waste images because CPU is very slow at training the custom YOLO model. A total of 3171 waste images were considered while experimenting on the Yolov3, Yolov3-tiny, Yolov4 and Yolov4-tiny models with the backbone as darknet53.conv.74, darknet19_448.conv.23, yolov4.conv.137 and Yolov4-tiny.conv.29. The network structure of the YOLO models was set up and trained to obtain the final weights of the custom YOLO object detector. The size of the final weight file was 240,680 Kilobyte and the size of the Yolov3 was 8.13 Kilobyte.

In conclusion, this article proposed Yolov3 with darknet53.conv.74 backbone as the state-of-the-art technology to automate waste object detection and classification at the source of the waste collection despite its limitation in speed of prediction. In addition, the KSA used to fine tune the learning rate of the network structure of Yolo can facilitate waste object detection. In the future, the custom YOLO object detector can be implemented on an IoT device and deployed on the design prototype proposed for waste bin and waste object collection.

## Figures and Tables

**Table 1:** Hardware specification of LattePanda alpha 864.

Specification	Description
CPU	Intel m3-8100Y
Graphics	Intel HD Graphics 615, 300–900 MHz
Memory	8 GB LPDDR3 RAM
Storage	64 GB
Connectivity	Wi-Fi 802.11AC 2.4 G & 5 G, Dual-Band Bluetooth 4.2, Gigabyte Ethernet
Display	4 K HDMI Output, Type-C, DP Support
Operating system	Windows 10 Pro
Dimensions	115 × 78 × 14 mm

**Table 2:** Classification of waste image datasets.

Class of Waste	Recyclable
Mixed paper (leaflet and brochure, newspaper)	Yes
Metal can/tin	Yes
Metallic foil	Yes
Glass bottle (colored and colorless)	Yes
Plastic garbage bag	Yes
Plastic bottle	Yes
Polystyrene	Yes
Snack plastic bag	Yes

**Table 3:** YOLO models and backbone.

YOLO Model	Number of Fully Connected YOLO Layer	Backbone
**Yolov3**	3	darknet53.conv.74
**Yolov3-tiny**	2	darknet19_448.conv.23
**Yolov4**	3	yolov4.conv.137
**Yolov4-tiny**	2	yolov4-tiny.conv.29

**Table 4:** Hyper-parameter for YOLO architecture.

YOLO Model	Batch	Mini-Batch	Learning Rate (Default)	Momentum (Default)	Decay (Default)
**Yolov3**	64	32	0.001	0.9	0.0005
**Yolov3-tiny**	64	32	0.001	0.9	0.0005
**Yolov4**	64	32	0.001	0.9	0.0005
**Yolov4-tiny**	64	16	0.0026	0.9	0.0005

**Table 5:** Performance result of different YOLO models on classes of waste dataset.

YOLO Models	Newspaper	Metal Cans/Tins	Metallic Foil	Glass Bottles	Plastic Garbage Bags	Plastic Bottles	Polystyrene	Snack Plastic Bag	Average Precision (AP)
Yolov3	97%	100%	100%	98%	100%	75%	99%	99%	96%
Yolov3-tiny	0	99%	30%	96%	0	47%	0	100%	47%
Yolov4	28%	57%	55%	40%	0	49%	33%	32%	37%
Yolov4-tiny	35%	72%	61%	70%	68%	52%	36%	54%	56%

**Table 6:** Performance result of different versions of YOLO model and class of a waste dataset.

	Yolov3					AP (%)
	Test Precision (%)					
Class of Waste	1	2	3	4	5	
Newspaper	96	88	85	95	95	92
Metal can/Tin	98	99	98	93	94	96
Metallic foil	97	72	95	87	97	90
Glass bottle	98	95	97	99	99	98
Plastic garbage bag	89	67	62	42	51	62
Plastic bottle	75	98	90	98	99	92
Polystyrene	99	48	44	64	69	65
Snack plastic bag	51	41	33	46	50	44
*mean AP (%)*						***80***
	**Yolov3-tiny**					**AP (%)**
	**Test precision (%)**					
**Class of waste**	**1**	**2**	**3**	**4**	**5**	
Newspaper	47	69	77	63	56	62
Metal can/Tin	83	92	90	45	87	79
Metallic foil	30	51	43	76	53	51
Glass bottle	96	67	38	43	44	58
Plastic garbage bag	38	32	36	45	47	40
Plastic bottle	86	96	93	78	65	84
Polystyrene	38	36	44	86	33	47
Snack plastic bag	41	42	34	30	35	36
*mean AP (%)*						57
	**Yolov4**					**AP (%)**
	**Test precision (%)**					
**Class of waste**	**1**	**2**	**3**	**4**	**5**	
Newspaper	37	45	52	54	49	47
Metal can/Tin	57	28	28	30	27	34
Metallic foil	50	51	57	58	52	54
Glass bottle (colored and colorless)	40	30	35	34	42	36
Plastic garbage bag	31	35	32	36	33	33
Plastic bottle	49	68	50	39	38	49
Polystyrene	33	35	34	31	36	34
Snack plastic bag	32	45	48	36	51	42
*mean AP (%)*						***41***
	**Yolov4-tiny**					**AP (%)**
	**Test precision (%)**					
**Class of waste**	**1**	**2**	**3**	**4**	**5**	
Newspaper	56	89	80	66	79	74
Metal can/Tin	85	86	85	87	56	80
Metallic foil	82	82	88	82	87	84
Glass bottle (colored and colorless)	88	93	76	79	83	84
Plastic garbage bag	80	88	89	88	86	86
Plastic bottle	86	83	86	79	85	84
Polystyrene	82	64	58	81	73	72
Snack plastic bag	19	20	33	26	36	27
*mean AP (%)*						***74***

**Table 7:** BFLOPS and number of layers loaded.

YOLO Models	Total BFLOPS	Layers Loaded from Trained File
Yolov3	65.355	107
Yolov3-tiny	5.459	24
Yolov4	127.341	162
Yolov4-tiny	6.798	38

**Table 8:** Class of waste image and speed of prediction (milli-seconds) on a test dataset.

YOLO Models	Newspaper	Metal Cans/Tins	Metallic Foil	Glass Bottles	Plastic Garbage Bags	Plastic Bottles	Polystyrene	Plastic Snack Bag	Average Speed
Yolov3	14,864.519	14,408.218	13,992.650	14,340.804	15,797.001	14,339.928	14,479.301	13,868.197	14,511.327
Yolov3-tiny	1713.719	1787.309	1718.016	1597.968	1751.314	1594.539	1760.403	1832.831	1719.5124
Yolov4	38,687.950	37,195.259	38,249.108	37,453.995	46,097.332	38,102.992	37,957.456	37,909.434	38,956.691
Yolov4-tiny	2387.265	2011.539	2106.241	2101.936	2191.841	2154.326	2353.303	2330.005	2204.557

**Table 9:** Average speed of prediction (milli-seconds) of Yolo models on a test dataset.

	Yolov3					
	Speed of Prediction (Milli-Seconds) for Each Test Precision					
Class of Waste	1	2	3	4	5	Average Speed
Newspaper	145,11.33	3371.072	3402.911	3423.648	3408.782	5623.548
Metal can/Tin	3374.501	3358.799	3361.697	3687.178	3396.21	3435.677
Metallic foil	4431.427	3366.969	3420.938	3450.327	3382.536	3610.439
Glass bottle	3259.033	3284.746	3241.885	3300.003	3235.94	3264.321
Plastic garbage bag	3229.904	3242.865	3288.991	3260.612	3220.934	3248.661
Plastic bottle	3248.995	3259.026	3285.452	3278.581	3242.654	3262.942
Polystyrene	3266.775	3252.742	3252.253	3298.082	3294.333	3272.837
Snack plastic bag	3243.03	3251.574	3269.722	3267.892	3260.797	3258.603
	**Yolov3-tiny**					
	**Speed of prediction (milli-seconds) for each Test precision**					
**Class of waste**	**1**	**2**	**3**	**4**	**5**	**Average Speed**
Newspaper	1719.5124	345.386	451.605	355.934	349.96	644.4795
Metal can/Tin	412.516	362.064	348.336	363.067	3,507,220	701,741.2
Metallic foil	364.925	366.693	349.6	347.747	355.349	356.8628
Glass bottle	361.354	361.13	375.465	354.422	352.705	361.0152
Plastic garbage bag	365.59	366.693	361.351	357.466	357.192	361.6584
Plastic bottle	350.41	349.458	356.926	354.744	352.455	352.7986
Polystyrene	347.004	345.738	347.331	358.453	347.524	349.21
Snack plastic bag	353.371	356.43	357.425	364.276	354.909	357.2822
	**Yolov4**					
	**Speed of prediction (milli-seconds) for each Test precision**					
**Class of waste**	**1**	**2**	**3**	**4**	**5**	**Average Speed**
Newspaper	38,956.691	8774.513	9017.872	8828.029	8830.348	14,881.49
Metal can/Tin	9064.939	8705.701	8840.907	8755.587	8809.887	8835.404
Metallic foil	8885.9	8734.941	8770.792	8712.028	8813.188	8783.37
Glass bottle	8927.729	8798.003	8646.429	8704.054	8642.384	8743.72
Plastic garbage bag	8785.54	8773.475	8792.293	8672.216	8569.058	8718.516
Plastic bottle	8678.185	8730.985	8727.078	8807.571	8711.908	8731.145
Polystyrene	8765.683	8796.807	8615.674	8900.68	8932.007	8802.17
Snack plastic bag	8741.221	8813.443	8908.464	8672.81	8762.721	8779.732
	**Yolov4-tiny**					
	**Speed of prediction (milli-seconds) for each Test precision**					
**Class of waste**	**1**	**2**	**3**	**4**	**5**	**Average Speed**
Newspaper	2204.557	432.81	444.677	433.673	425.816	788.3066
Metal can/Tin	506.594	461.378	438.879	434.285	429.553	454.1378
Metallic foil	538.672	501.698	427.958	436.49	438.808	468.7252
Glass bottle	466.04	471.653	450.824	460.716	447.763	459.3992
Plastic garbage bag	446.845	439.637	451.61	440.731	431.29	442.0226
Plastic bottle	444.43	483.175	439.567	428.941	427.13	444.6486
Polystyrene	515.868	489.658	429.741	425.873	431.69	458.566
Snack plastic bag	432.811	435.474	431.187	426.308	446.415	434.439

**Table 10:** KSA learning rate parameter.

#No	Iteration#1	Iteration#2	Iteration#3	Iteration#4	Iteration#5
1	0.0008	0.0028	0.0045	0.0099	0.0124
2	0.0109	0.0111	0.0021	0.0678	0.0689
3	0.0210	0.0234	**0.0009**	0.0987	0.0897
4	0.1002	**0.0009**	0.0045	0.0123	**0.0009**
5	0.0028	0.0070	0.0032	**0.0007**	0.0291

Bold values represent the minimum learning rate parameter in each column

**Table 11:** Performance result of KSA-based YOLO model on class of waste dataset.

YOLO Models	Newspaper	Metal Cans/Tins	Metallic Foil	Glass Bottles	Plastic Garbage Bags	Plastic Bottles	Polystyrene	Snack Plastic Bag	AP (%)
Yolov3	96%	98%	99%	98%	99%	78%	99%	98%	96%
Yolov3-tiny	50%	97%	35%	95%	30%	48%	40%	95%	61%
Yolov4	30%	60%	56%	44%	27%	50%	38%	30%	42%
Yolov4-tiny	40%	75%	66%	74%	69%	55%	39%	50%	59%

## Data Availability

Dataset used for training is available on: https://doi.org/10.6084/m9.figshare.20427696.v2 (accessed on 5 August 2022).

## Abbreviations

AI	Artificial Intelligence
ANN	Artificial Neural Networks
ATD	Automated Teller Dustbin
BFLOPS	Billion Floating Point Operation Per Second
CNN or ConvNet	Convolutional Neural Network
MS COCO	Microsoft Common Object in Context
CPU	Central Processing Unit
DTW	Dynamic Time Warping
EdgeIoT	Edge computing Internet of Things
GPU	Graphics Processing Unit
GW	Gabor Wavelet
IoT	Internet of Things
IOU	Intersection Over Union
MLP	Multi-Layer Perceptron
R-CNN	Region proposal CNN
RFID	Radio Frequency Identification
RNN	Recurrent Neural Network
SVM	Support Vector Machine
SAUoT	South African University of Technology
YOLO	You-Only-Look-Once
KSA	Kestrel-based Search Algorithm
DRL	Deep Reinforcement Learning
DQLN	Deep Q-Learning Network
LED	Light Emitting Diode
BBoxes	Bounding Boxes
ROI	Region of Interest
MTTP	Maximum Power Point Tracker
IDE	Integrated Development Environment
AP	Average Precision
